# Evaluating Providers’ Prescription Opioid Instructions to Pediatric Patients

**DOI:** 10.3390/children9050707

**Published:** 2022-05-11

**Authors:** Denise D. Tran, Patrick C. M. Brown, Corrin Murphy, Diana Ho, Karen A. Hudson, Anna C. Wilson, Sarah W. Feldstein Ewing

**Affiliations:** 1 Keck School of Medicine, University of Southern California, Los Angeles, CA 90033, USA; denise.tran@med.usc.edu; 2 Department of Pediatrics, Oregon Health & Science University, Portland, OR 97239, USA; brownpat@ohsu.edu (P.C.M.B.); murpcor@ohsu.edu (C.M.); longann@ohsu.edu (A.C.W.); 3 Department of Psychology, University of Rhode Island, Kingston, RI 02881, USA; hodiana@uri.edu (D.H.); hudsonk@uri.edu (K.A.H.)

**Keywords:** pediatric, children/adolescents, pediatric opioids, medication instructions

## Abstract

Receiving an opioid prescription during childhood increases the risk of hazardous prescription opioid (PO) use during emerging adulthood. Instruction on how to safely use POs plays an essential role in pediatric patients’ capacity to utilize as well as to discontinue POs appropriately. This study aimed to evaluate pediatric PO label instructions provided to a large sample of pediatric outpatients. Data were extracted from the electronic healthcare records system identifying pediatric patients who received a PO between 2016 and 2019 from pediatric outpatient medical clinics were affiliated with a northwestern United States medical center and children’s hospital. Pediatric patients (n = 12,613) between 0–17 years old who received a PO during outpatient care were included. Patients with chronic health conditions (e.g., cancer) or who received their PO from an inpatient medical setting were excluded. Patient demographics, medication instructions, associated diagnoses, and other prescription information (e.g., name of medication, dose, and quantity dispensed) were examined using automated text classification. Many label instructions did not include any indication/reason for use (20.8%). Virtually none of the POs (>99%) included instructions for how to reduce/wean off POs, contact information for questions about the POs, and/or instructions around how to dispose of the POs. Efforts are needed to ensure that pediatric PO instructions contain essential elements to improve comprehension of when and how to use POs for pediatric patients.

## 1. Introduction

Prescription opioids (POs) given to children during the course of routine outpatient treatment for pain can increase risk for hazardous PO use during young adulthood [1]. Childhood is an especially vulnerable period, as the use of POs before age 13 increases risk for later opioid use disorders (OUD) compared with later exposure [2]. Even the receipt of a PO for pain may unintentionally lead to pleasurable sensations of feeling “high” [3], and PO prescriptions are associated with a 33% increased risk for later hazardous PO use among children who would otherwise be at low risk for OUD (e.g., no history of substance use) [1].

Many adolescents are first introduced to POs through legitimate prescriptions to treat pain by medical providers [1,4]. No formal guidelines exist for PO prescribing for acute pain in children. Although opioids are now used less in clinical practice where possible, opioids are still necessary to control pain in some cases, particularly post-operatively [5]. The rates of adolescent PO use are high, with 7.2% and 14.3% of high school students reporting current and lifetime PO use, respectively [6]. Additionally, the rates of PO-related suicides in this age group continue to increase [7,8]. This is concerning given that medical providers tend to prescribe more POs than medically necessary for pediatric patients [9,10], leaving 58–92% of pediatric POs unused and available for potential hazardous use [10,11]. Further, only 1 in 5 families are informed about how to safely dispose of their children’s leftover POs [12].

Medication instructions play an essential role in parents′ management of pediatric medications and represent a key modifiable target within the domain of “opioid stewardship.” Specifically, when instructions are vague, they can be misinterpreted, leading to confusion and incorrect use of pediatric PO medications [13,14,15]. Awkward phrasing of instructions (e.g., 1 mg/1 mL solution—give 0.8 mg every 3 h prn pain) can be difficult to interpret, particularly for families who are not primary English speakers and/or who have lower levels of health literacy [16,17]. While pediatric providers have been urged to present instructions in a clear, concise, and unambiguous manner [14], these efforts have not always been successful.

Adult studies show that high levels of instruction complexity, specifically related to dosing, have been associated with greater confusion or error in medication use; medication labels with multistep instructions have been reported to be more difficult to use as prescribed [18]. Prior studies have also found that dosing instructions with less complexity, such as those using numerals (“1” vs. “one”) and simple medication descriptions (“pill” vs. “tablet”), were preferred by adult patients [19]. “Take-Wait-Stop” labeling, which simplifies text (e.g., replacing “do not exceed” with “do not take more than”) and separates instructions more clearly into the number of pills to take, minimum interval between doses, and maximum daily dose, reduced errors in adult PO medication use [20]. Specifying “time periods” instead of “times per day” and specific times (e.g., take at bedtime) in place of hourly intervals (e.g., take every 4–6 h) also enhanced adult comprehension in medication use [21]. 

More data are needed to understand the nature of pediatric PO instructions for young patients. Complicating these instructions, the vast majority of pediatric PO instructions specify that medication is to be taken “as needed”, but many fail to include how, when, or why to use and how, when, or why to discontinue use [22,23]. Additionally, while parents may receive additional written discharge or after-visit instructions on paper or in electronic communication, these often contain an overwhelming amount of text and are frequently either misplaced and/or not kept with the medication [24]. This leaves parents with little information about how to safely use and stop or taper pediatric PO use as their child’s pain resolves. In this exploratory study, we examined label instructions for pediatric PO use given during routine outpatient pain management. Specifically, we aimed to describe the structure and content of pediatric PO instructions with a young sample. We also aimed to determine whether the characteristics of the pediatric PO label instructions differed based on the prescribing department and age of the pediatric patient. We hypothesized that pediatric PO medication instructions would differ between surgical and medical specialties due to different workflows and indications (painful condition vs. post-operative pain) and between older and younger patients due to different provider perceptions of patient autonomy and comprehension.

## 2. Methods

### 2.1. Procedures

Data were collected using the Research Data Warehouse (RDW), a service provided by the Oregon Clinical and Translational Research Institute (OCTRI) at a northwest medical school. The RDW provided a repository of data from the electronic medical records of pediatric patients who were eligible for the study. Prescription information for children ages 0 to 17 years who received a pediatric PO between 1 January 2016 and 31 December 2019 in the context of routine pediatric outpatient or ambulatory care met inclusion criteria and was extracted from the RDW. Information related to patient demographics, type of PO, dose, quantity dispensed, instructions provided on the prescription label, and associated diagnoses were extracted. In order to capture pediatric POs given in outpatient settings for generally healthy children, patients with chronic diseases (e.g., cystic fibrosis), patients with blood disorders or cancer/neoplasms, patients with congenital disorders, those who received their pediatric PO through an inpatient unit, and routes of administration other than oral were excluded. This investigation was approved by the participating Institutional Review Board. 

### 2.2. Classification of Text Features and Data Processing

**Figure 1:**
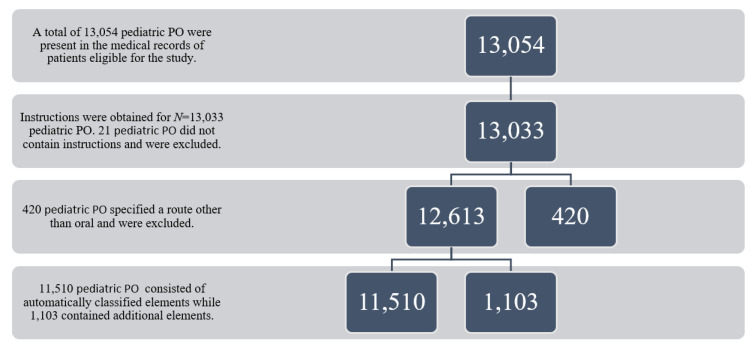
Flowchart of pediatric prescription opioid (PO) instructions examined.

Pediatric PO instruction features posited to impact instruction clarity (e.g., specific dosing instructions, maximum amount of medication to use per day, and when to discontinue use) were identified and included (Figure 1). An initial pediatric PO instruction review was performed to identify commonly used syntax structures. The ‘tidyverse’, ‘textclean’, and ‘dplyr’ packages in RStudio (Version 1.3.1093) automatically segmented and classified the pediatric PO instructions’ field elements into the following categories: (1) initial verb, (2) whether the amount of pediatric PO medication to be given at one time was present as a range or discrete quantity, (3) whether frequency with which to give the pediatric PO medication was present as a range or discrete frequency, (4) whether pediatric PO quantity to be given was expressed per day or as an hourly frequency, (5) route of pediatric PO administration, (6) reason for pediatric PO use or indication (e.g., “for pain”), (7) pediatric PO instructions stating to give as needed, (8) potentially confusing phrasing in pediatric PO instructions, and (9) any additional pediatric PO instructions. Potentially confusing language included terms not commonly used to describe pain, such as pain “refractory to” or “uncontrolled by” another medication or “multimodal” pain control.

An iterative process was used to refine classification by identifying frequently repeated pediatric PO phrases not automatically classified in the preceding round and including them in the algorithm for the subsequent round; an additional 5% of pediatric POs were manually classified to verify the accuracy of automatic classification. Elements that were not automatically classified in the preceding process were reviewed by two study team members. 

### 2.3. Statistical Analysis

Analyses were performed in RStudio (Version 1.3.1093). Pediatric PO instructions specifying a route of pediatric PO administration other than oral were excluded from analysis as these are less representative of the low complexity pediatric ambulatory care population examined in this study. Frequency tables were developed for pediatric PO instruction features posited to impact instruction clarity (e.g., specific dosing instructions, maximum amount of medication to use per day, when to discontinue use), as well as elements that occurred frequently during the iterative review of pediatric PO instructions by study team members. 

Frequency of each pediatric PO instruction element was compared between surgical and non-surgical prescribing departments and between patients 13 and older and those younger than 13 for those pediatric PO-instruction elements with significant variation across pediatric PO prescriptions (>5% in each category). Chi-square tests were used for these analyses. The odds ratios (ORs) and 95% confidence intervals (CIs) were calculated.

Surgical departments included those specialties recognized by the American College of Surgeons: cardiothoracic surgery, colon and rectal surgery, general surgery, gynecology and obstetrics, gynecologic oncology, neurological surgery, ophthalmic surgery, oral and maxillofacial surgery, orthopedic surgery, otorhinolaryngology, pediatric surgery, plastic and maxillofacial surgery, urology, and vascular surgery. All other departments were classified as non-surgical. Non-surgical departments included outpatient general and specialty clinics.

## 3. Results

### 3.1. Pediatric PO Patient Characteristics

A total of N = 11,213 pediatric PO patients ages 0–17 were identified. In line with this metropolitan region, this sample was predominately non-Hispanic white (78%), English-speaking (91.1%), with a mean age of 11.1 (SD = 5.5), and N = 2032 (18.1%) Hispanic. In addition, N = 6563 (58.5%) pediatric PO patients were male and N = 4650 (41.4%) were female (see Table 1).

### 3.2. Pediatric PO Instructions

Characteristics of pediatric PO instructions are listed in Table 2. Pediatric PO instructions given from a surgical department were significantly less likely to specify a discrete amount of pediatric PO medication to take at one time (OR = 0.77, 95% CI [0.70, 0.85], *p* < 0.001) and less likely to contain potentially confusing language (OR = 0.77, 95% CI [0.70, 0.86], *p* < 0.001) than pediatric PO instructions from a non-surgical department were (Table 3). Pediatric PO instructions from a surgical department were significantly more likely to contain pediatric PO instructions to limit use (OR = 1.74, 95% CI [1.53, 1.97], *p* < 0.001) and to specify the pain severity for which pediatric PO use was intended (OR = 2.72, 95% CI [2.45, 3.02], *p* < 0.001) compared with pediatric PO instructions from a non-surgical department.

Pediatric PO instructions written for patients who were at least 13 years old were significantly more likely to specify a discrete amount of pediatric PO to take at one time (OR = 2.35, 95% CI [2.15, 2.57], *p* < 0.001) and more likely to contain pediatric PO instructions to limit use (OR = 1.20, 95% CI [1.05, 1.37], *p* < 0.001) than pediatric PO instructions written for patients under 13 years old (Table 3). Pediatric PO instructions written for patients who were at least 13 years old were significantly less likely to contain potentially confusing language (OR = 0.78, 95% CI [0.71, 0.86], *p* < 0.001) than pediatric PO instructions written for patients under age 13. There was no significant difference in the frequency with which pediatric PO instructions specified the pain severity for which the pediatric PO use was intended between these age groups (OR = 1.02, 95% CI [0.94, 1.11], *p* > 0.05). 

## 4. Discussion

Our team was not able to identify any peer-reviewed published manuscripts that utilized an empirical approach to examine pediatric PO medication label instructions provided to young patients and their caregivers; thus, our study contributes novel empirical information about label instructions provided for children receiving POs. In our study, among the 12,613 pediatric PO label instructions given to young patients in the course of routine outpatient treatment, a number of beneficial elements were identified. For example, 98.6% of the pediatric PO instructions specified a frequency with which the PO should be taken (e.g., “every # hours”), and 99.4% included a route of administration. An amount of pediatric PO to take per day was observed in 99.4% of instructions, and among them, 75.8% contained a specified discrete amount (e.g., “2 tablets”) rather than a range (e.g., “5 to 10 mL” or “1–2 pills”). Lastly, an indication (e.g., “for pain”) was specified in 78.9% of the instructions, and among these, 89.7% included a severity level (e.g., for moderate or severe pain). Indications were considered highly important for safe pediatric PO use by supporting pediatric patients’ and their families’ understanding of the steps necessary to take the pediatric PO in the manner that the provider intended [25].

Areas for pediatric PO improvement were also identified. For example, 21.1% pediatric PO labels did not include an indication, and 14.9% included a range (e.g., 2–4 tablets) rather than a specific amount to take (e.g., 3 tablets). Further, there were no clear directions on when to use and how and when to discontinue use. The vast majority (98.1%) instructed children with pain to take “as needed” rather than including specific instructions on when pediatric POs should be taken or in what situations/circumstances the pediatric patient and/or parent should consider the pediatric PO as being medically “needed”. Most (98.9%) pediatric PO instructions did not contain directions regarding when to discontinue or wean off of the pediatric PO, and among the minority of instructions that mentioned weaning (*n* = 142), only 68 (0.5%) included specific instructions as to *how* to wean. Only 3.5% included a maximum total amount of pediatric PO to take, and 3.2% included the maximum duration the pediatric PO should be taken. Increasing the rate at which prescribers include these elements might be beneficial for patient and parent understanding of how and when to safely discontinue. Thus, these data indicate that major areas of improvement include using more specific instructions on the amount of pediatric PO to take, providing clearer directions on when or in what situations/circumstances to use, and how to wean off of or discontinue. 

Potentially confusing phrasing in pediatric PO instructions were common, with 19.4% containing medical jargon (e.g., “breakthrough pain”, “when tolerating po only”), requiring a higher reading level to comprehend (e.g., “anticipatory”, “ameliorate”, “multimodal”), or for previously unspecified but clearly confusing text (e.g., “for severe pain >8/10”). Zheng et al. [26] reported that 11.3% of e-prescription directions contained at least one quality control issue even after being transcribed by pharmacy staff. In line with our findings, Zheng et al. reported similar variability, complexity, and ambiguity, including instructions containing abbreviations such as ‘tab’ and ‘po’ (per os). While it is clear that prescribing physicians are not trying to set the stage for future OUD, the experience of using pediatric POs even exactly as prescribed can orient the brain and behavior to anxiolytic as well as pain-reduction effects, which can set the stage for future hazardous PO use [27]. 

It can be especially challenging for patients to interpret and follow the pediatric PO instructions when they contain abbreviations or medical jargon, and this may even be more pronounced for less formally-educated families and/or for families for whom English is not their first language. The average U.S. adult reads at an eighth-grade level [28]. Given U.S. adult reading skills, the American Medical Association (AMA) and National Institutes of Health (NIH) officially advise that patient materials not exceed the sixth-grade reading level [29,30]. Therefore, removal of medical jargon and creating labels with more simple and straightforward pediatric PO instructions should aid patients in comprehension and would not disadvantage those with lower literacy rates [21,31]. 

Additionally, contact information for families’ follow-up questions were provided in fewer than 0.001% of the pediatric PO instructions; this is highly problematic given the need for additional information/clarification from the prescribing provider. While families may have this information in other locations (e.g., in printed or electronic patient instructions), making this information easily accessible may be helpful for increasing patient–provider communication around pediatric PO use and discontinuation. Similarly, fewer than 0.001% of pediatric PO labels included instructions on how to dispose of leftover pediatric POs. In fact, recent findings indicate that nearly half of adolescents who use POs in hazardous ways receive them from friends and relatives [32], suggesting the importance of reducing leftover pediatric POs by including clear disposal instructions. Including clear instructions on how to dispose of leftover pediatric POs can decrease potential availability of pediatric POs for future hazardous PO use. 

Results of analyses indicate that patients 13 years of age and older were more likely to receive more precise pediatric PO instructions and less likely to receive potentially confusing instructions than patients younger than age 13. Prior research indicates that 25% of individuals who were prescribed opioids at 13 years or younger may be at enhanced risk for transitioning into OUD [1]. Therefore, it may be particularly important to improve the instructions provided to young patients, particularly those under age 13, to mitigate risk for hazardous use. Compared with non-surgical departments, surgical departments provided significantly fewer pediatric PO instructions that contained specific dosing instructions. However, surgical departments had significantly more instructions that directed patients to limit their pediatric PO use and contained more context as to what the pediatric PO should be used for. Despite significant differences, only 12.8% and 7.8% of pediatric PO instructions provided by surgical and non-surgical departments, respectively, contained directions to limit use, while 16.6% and 20.5% of instructions contained potentially confusing language. These findings represent several areas for continued improvement in pediatric PO prescribing practices for providers working with young patients and their families. Here as well, removal of potentially confusing language in pediatric PO instructions and providing directions to limit use can aid in patient comprehension and reduce inappropriate pediatric PO use.

## 5. Limitations and Future Directions

Our study has numerous strengths, including a careful empirical evaluation. At the same time, results should be interpreted in light of the following limitations. Because data were extracted from medical records of pediatric patients who received a pediatric PO, information about other variables of interest could not be evaluated. For example, future research may examine whether provider demographics and other patient characteristics are associated with the quality of written pediatric PO instructions provided, given that these characteristics can exacerbate problems in patient–provider interactions, quality of care, treatment adherence, and continuity of care [33]. Further, future research may also examine how differences in prescribing practices may contribute to these differences and other PO-related health disparities. Due to a low percentage (<20%) of prescriptions having an associated diagnosis in our data, we were also unable to look at the diagnoses for which these prescriptions were received. Future studies could identify these by linking prescription data to encounter data.

## 6. Conclusions

Addressing the ambiguity, technicality, and variability of pediatric PO label instructions is among one of the most actionable avenues to improve comprehension around safe pediatric PO use by young people and their families. Increased efforts to develop structured systems or tools to help standardize label directions, such as embedding a comprehensive set of direction components into the electronic health record, could improve the quality of these directions and potentially increase appropriate pediatric PO use by patients [26]. In addition to producing clearer directions, instructions should be provided at an appropriate reading level without medical jargon and abbreviations as well as contain contact information for follow-up questions, specific weaning and termination directions, and steps to dispose of unused pediatric POs.

## Figures and Tables

**Table 1:** Demographic information of study sample.

	*N* (%)	*M* (*SD*)
Age		11.1 (5.5)
Gender		
Female	4650 (41.5%)	
Male	6563 (58.5%)	
Race		
non-Hispanic White	8747 (78.0%)	
African American	255 (2.3%)	
Asian	355 (3.2%)	
Multiracial	916 (8.2%)	
Unknown or not reported	940 (8.4%)	
Ethnicity		
Hispanic	2032 (18.1%)	
Not Hispanic	8625 (79.6%)	
Unknown or not reported	556 (5.0%)	
Primary language		
English	10,219 (91.1%)	
Spanish	733 (6.5%)	
Other	169 (1.6%)	
Unknown	92 (0.8%)	

**Table 2:** Characteristics of pediatric prescription opioid (PO) instructions.

	*N* (%) with element
Amount of pediatric PO to take	12,538 (99.4%)
Discrete amount	10,664 (85.1%)
Range	2474 (14.9%)
Amount as numeral	12,508 (99.8%)
Amount as word	30 (0.2%)
Frequency of pediatric PO to take	12,542 (99.4%)
Specified frequency (every # hours)	12,365 (98.6%)
Range (every # hours)	107 (0.9%)
Specified frequency (# per day)	69 (0.6%)
Range (# per day)	1 (0.0%)
Route of pediatric PO administration	12,534 (99.4%)
“As needed”	12,369 (98.1%)
Pediatric PO indication specified	9954 (78.9%)
Severity of pain specified	9171 (92.1%)
Cause or location of pain specified	235 (2.4%)
Additional pediatric PO instructions	1534 (12.2%)
Instructions to limit pediatric PO medication	1161 (9.2%)
Maximum dosing frequency	12 (1.0%)
Maximum total amount	477 (38.5%)
Maximum duration for pediatric PO use	400 (34.5%)
Direction to use non-PO medication first	304 (26.2%)
Instruction to minimize amount of pediatric PO	19 (1.6%)
Instructions to wean pediatric PO	142 (1.1%)
Weaning steps specified for pediatric PO	68 (47.9%)
Potentially confusing phrasing	2443 (19.4%)

*Note.* A total of 12,613 pediatric PO were analyzed.

**Table 3:** Results of analytic comparisons.

	Prescribing Department				Patient Age			
Characteristic	Surgical	Non-Surgical	* OR *	95% CI	≥13 Years	<13 Years	*OR*	95% CI
N (%) with specified discrete amount of pediatric PO	2714 (76.6%)	7349 (81.0%)	0.77 ***	[0.70, 0.85]	3027 (70.3%)	7037 (84.7%)	2.35 ***	[2.15, 2.57]
N (%) with instructions to limit pediatric PO	454 (12.8%)	707 (7.8%)	1.74 ***	[1.53, 1.97]	354 (15.3%)	807 (9.7%)	1.20 ***	[1.05, 1.37]
N (%) with severity of pain specified	3016 (85.1%)	6154 (67.9%)	2.72 ***	[2.45, 3.02]	3122 (72.4%)	6049 (72.8%)	1.02	[0.94, 1.11]
N (%) with potentially confusing phrasing on pediatric PO	587 (16.6%)	1856 (20.5%)	0.77 ***	[0.70, 0.86]	945 (21.9%)	1498 (18.0%)	0.78 ***	[0.71, 0.86]

*Note*. *OR* = Odds ratios; CI = confidence interval. *** *p* < 0.001.

## Data Availability

Restrictions apply to the availability of these data. Data were obtained from OCTRI and are available from the author team with the permission of Drs. Sarah W. Feldstein Ewing and Anna Wilson.
